# Comparative chloroplast genome and phylogenetic analyses of Chinese *Polyspora*

**DOI:** 10.1038/s41598-022-16290-4

**Published:** 2022-09-26

**Authors:** Zhi-Feng Fan, Chang-Le Ma

**Affiliations:** 1 Southwest Research Center for Engineering Technology of Landscape Architecture (State Forestry and Grassland Administration), College of Landscape Architecture and Horticulture Sciences, Southwest Forestry University, Kunming, 650224 People’s Republic of China; 2 Kunming University of Science and Technology, Kunming, 650500 People’s Republic of China

**Keywords:** Evolution, Genetics, Molecular biology, Plant sciences

## Abstract

*Polyspora* Sweet (Theaceae) are winter ornamental landscape plants native to southern and southeastern Asia, some of which have medicinal value. The chloroplast (cp) genome data of *Polyspora* are scarce, and the gene evolution and interspecific relationship are still unclear. In this study, we sequenced and annotated *Polyspora chrysandra* cp genome and combined it with previously published genomes for other Chinese *Polyspora* species. The results showed that cp genomes of six Chinese *Polyspora* varied in length between 156,452 bp (*P. chrysandra*) and 157,066 bp (*P. speciosa*), but all contained 132 genes, with GC content of 37.3%, and highly similar genes distribution and codon usage. A total of eleven intergenic spacer regions were found having the highest levels of divergence, and eight divergence hotspots were identified as molecular markers for Phylogeography and genetic diversity studies in *Polyspora*. Gene selection pressure suggested that five genes were subjected to positive selection. Phylogenetic relationships among *Polyspora* species based on the complete cp genomes were supported strongly, indicating that the cp genomes have the potential to be used as super barcodes for further analysis of the phylogeny of the entire genus. The cp genomes of Chinese *Polyspora* species will provide valuable information for species identification, molecular breeding and evolutionary analysis of genus *Polyspora*.

*Polyspora* Sweet is a genus of 48 accepted species in Theaceae family which are distributed in subtropical and tropical regions of southern and southeastern Asia^1,2^. Most species in *Polyspora* have high ornamental value with beautiful canopy shape and pretty flowers blooming in winter, and can be used as landscape trees^3–5^. Some of them have edible and medicinal value because of the natural antioxidants in their fruits^4,6,7^. Moreover, extracts from roots and stems may have cytotoxic activities^8–10^. Within Theaceae, analyses of chloroplast protein coding genes, mitochondrial gene and nuclear gene sequences suggest that *Polyspora* and *Camellia* are closely related and both belong to Theeae^1,11^. However, *Gordonia*, which has similar seed morphological characteristics as *Polyspora*, locates in Gordonieae. Although *Polyspora* have important horticultural and medicinal value, molecular data for phylogeography and genetic diversity studies are still limited.

Chloroplasts (cp) are organelles of photosynthesis in green plants, and also the important place for the synthesis of pigment, protein and starch^12,13^. In angiosperms, most cp genomes are usually inherited from maternal parents and conserved with stable structure and low genetic recombination rate^14^. The cp genomes of terrestrial plants are usually between 120–160 kb in length and have a tetrad structure: one large single copy (LSC), one small single copy (SSC) and two inverted repeats (IRs)^15–17^. The cp genome consists of 120–130 genes, mainly involved in photosynthesis, transcription, and translation^18^. Compared with nuclear and mitochondrial genomes, cp genomes are relatively small and rarely recombined^19,20^. Structural differences in cp genomes among related species are valuable for species identification^21^. Therefore, the complete cp genomes have become an ideal model for evolutionary and comparative genomic studies, which can provide important basis for revealing the phylogenetic and evolutionary relationships of phytogroup^22^. Though some researchers had used cp genome data to explore the phylogenetic relationships of some species in *Polyspora*^11,23,24^, the selected DNA fragments were few, and the structural characteristics of the whole cp genome had not been analyzed in greater detail.

In flora of China, there are 6 species of *Polyspora*, the cp genomes for five of them have been published previously, including *P. axillaris*, *P. speciosa*, *P. hainanensis*, *P. longicarpa* and *P. tiantangensis*. Most of *Polyspora* species are trees, while the two biotypes of tree and shrub coexist in *P. chrysandra*. The difference between calyx and petals of *P. chrysandra* is less obvious than that of other *Polyspora* species, which may be intermediate morphology of plant evolution in the genus. Here, we reported the complete cp genome sequence of *P*. *chrysandra*, and compared its sequence features with other five Chinese *Polyspora* species. The objective of this study were to: (1) characterize and compare the cp genomes of Chinese *Polyspora*, and detect differences among the six species; (2) identify repeat sequences, simple sequence repeats and genetically variable regions, select divergence hotpots as candidate DNA markers; (3) explore their IR expansion and contraction, estimate genes selective pressure and codon usage; (4) reconstruct phylogenetic relationships of *Polyspora* species based on the cp genome alignments and verify their phylogenetic position within Theaceae. Comprehensive chloroplast genomic analysis of Chinese *Polyspora* will provide fundamental basis for further understanding of the evolution of Theaceae family.

## Results

### Chloroplast genome structures and features of Polyspora species

**Figure 1:**
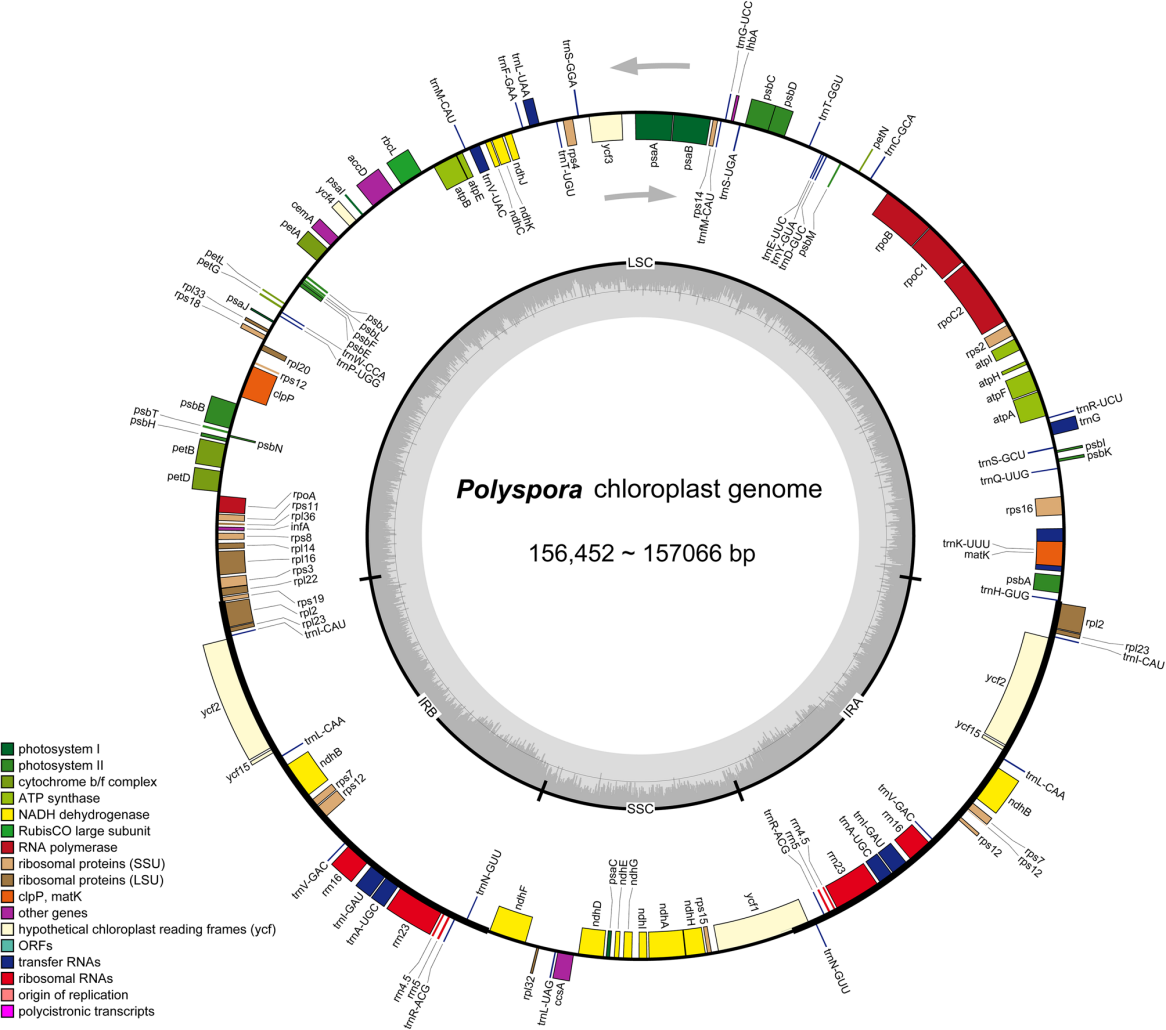
Chloroplast genome map of Chinese *Polyspora*. Genes on the outside of the circle are transcribed counterclockwise, while those inside are transcribed clockwise. Genes belonging to different functional groups are color-coded. Dark gray shading in the inner circle indicates guanine-cytosine content.

The complete cp genome of *P. chrysandra* was 156,452 bp in length, and was slightly less than other Chinese *Polyspora* species. It contained a LSC region of 85,924 bp, a SSC region of 18,318 bp, and two IR regions of 26,105 bp each. The cp genomes of six Chinese *Polyspora* species varied in length between 156,452 bp (*P. chrysandra*) and 157,066 bp (*P. speciosa*) (Fig. 1). The differences between the lengths were no greater than 614 bp. All six *Polyspora* cp genomes displayed a typical quadripartite structure, including a pair of IRs (26,077–26,105 bp) separated by one LSC (85,924–86,597 bp) and one SSC (18,284–18,318 bp) region. The GC content of all cp genomes were 37.3%. The cp genomes of Chinese *Polyspora* species showed similar genome structures, containing 132 genes, and comprised of 87 protein-coding, 37 tRNA, and 8 rRNA genes (Table S2). Among them, 18 genes were duplicated in the IR region, including 7 tRNA genes, 4 rRNA genes and 7 coding genes. There were 15 genes with one intron, including 6 tRNAs (*trnA-UGC*, *trnG*, *trnI-GAU*, *trnK-UUU*, *trnL-UAA*, *trnV-UAC*) and 9 coding genes (*atpF*, *ndhA*, *ndhB*, *petB*, *petD*, *rps16*, *rpl16*, *rpl2*, *rpoC1*). Three coding genes (*clpP*, *rps12*, and *ycf3*) had two introns (Table 1).

**Table 1:** Genes identified in the chloroplast genome of *Polyspora* species.

Category	Group	Gene name
Genes for photosynthesis	ATP synthase	*atpA*, *atpB*, *atpE*, atpF*, *atpH*, *atpI*
	ATP-dependent protease	clpP**
	NADH-dehydrogenase	ndhA*, *ndhB*(× 2)*, *ndhC*, *ndhD*, *ndhE*, *ndhF*, *ndhG*, *ndhH*, *ndhI*, *ndhJ*, *ndhK*
	Cytochrome b/f complex	*petA*, petB*, petD*, *petG*, *petL*, *petN*
	Photosystem I	*psaA*, *psaB*, *psaC*, *psaI*, *psaJ*
	Photosystem II	*psbA*, *psbB*, *psbC*, *psbD*, *psbE*, *psbF*, *psbH*, *psbI*, *psbJ*, *psbK*, *psbL*, *psbM*, *psbN*, *psbT*
	RubiscoCO large subunit	*rbcL*
Expression-related genes	Ribosomal RNA	*rrn16*(× 2), *rrn23*(× 2), *rrn4.5*(× 2), *rrn5*(× 2)
	transfer RNA	*trnA-UGC*(× 2)*, *trnC-GCA*, *trnD-GUC*, *trnE-UUC*, *trnF-GAA*, *trnfM-CAU*, trnG*, *trnG-UCC*, *trnH-GUG*, *trnI-CAU*(× 2), *trnI-GAU*(× 2)*, trnK-UUU*, *trnL-CAA*(× 2), trnL-UAA*, *trnL-UAG*, *trnM-CAU*, *trnN-GUU*(× 2), *trnP-UGG*, *trnQ-UUG*, *trnR-ACG*(× 2), *trnR-UCU*, *trnS-GCU*, *trnS-GGA*, *trnS-UGA*, *trnT-GGU*, *trnT-UGU*, *trnV-GAC*(× 2), trnV-UAC*, *trnW-CCA*, *trnY-GUA*
	Ribosomal protein (SSU)	*rps11*, *rps12*(× 2)*, *rps14*, *rps15*, rps16*, *rps18*, *rps19*, *rps2*, *rps3*, *rps4*, *rps7*(× 2), *rps8*
	Ribosomal protein (LSU)	*rpl14*, rpl16*, *rpl2*(× 2)*, *rpl20*, *rpl22*, *rpl23*(× 2), *rpl32*, *rpl33*, *rpl36*
	RNA polymerase	*rpoA*, *rpoB*, rpoC1*, *rpoC2*
Other genes	Maturase	*matK*
	Envelop membrane protein	*cemA*
	Subunit of acetyl-CoA-carboxylase	*accD*
	c-type cytochrome synthesis	*ccsA*
	Translation initiation factor IF-1	*infA*
Genes of unkown function	Hypothetical chloroplast reading frames	*ycf1*, *ycf15*(× 2), *ycf2*(× 2), ycf3**, *ycf4*, *lhbA*

*Indicates gene with one intron and **indicates gene with two introns; (× 2) indicates that the number of repeated units is 2.

### Expansion and contraction of inverted repeats

**Figure 2:**
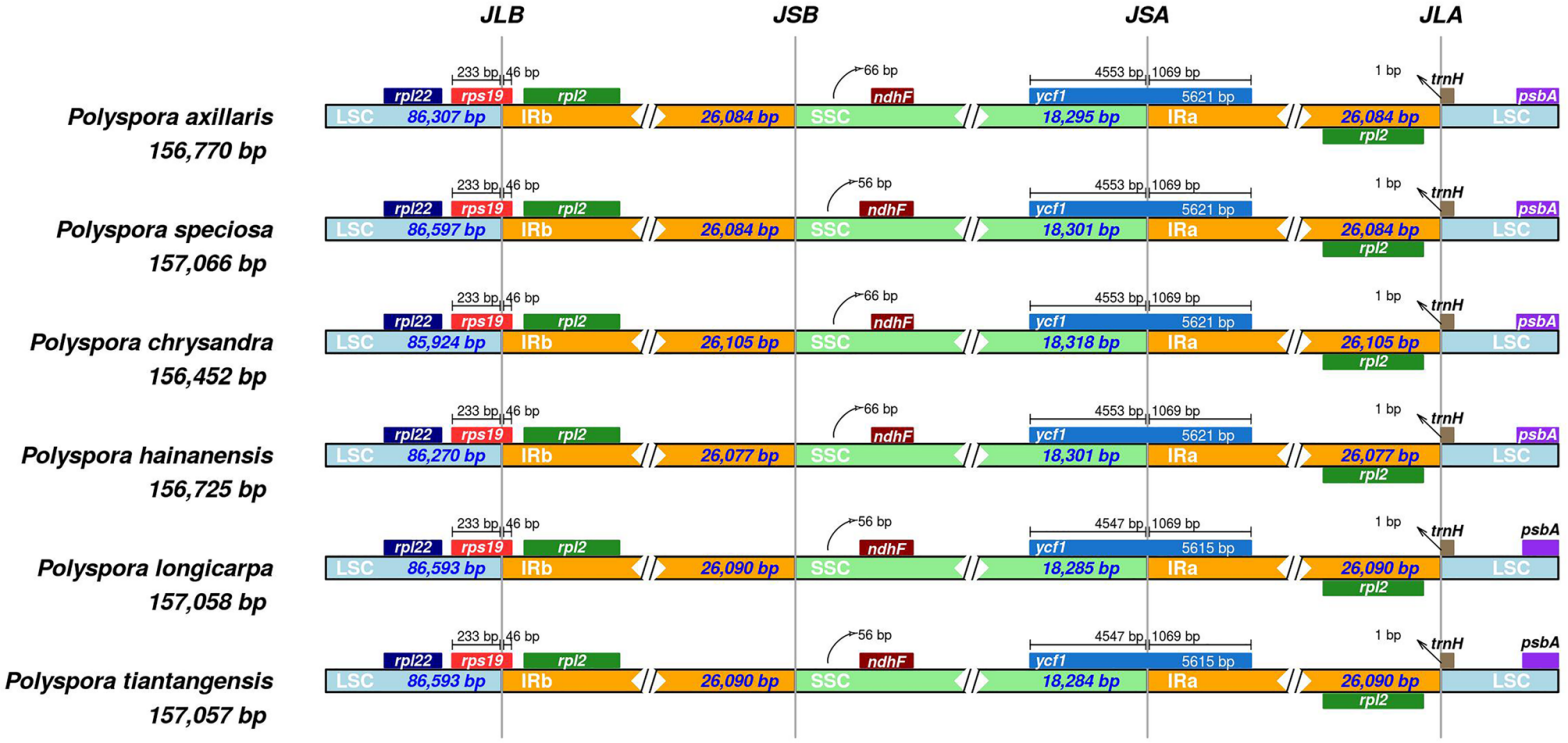
Comparison of the border regions of the cp genomes among Chinese *Polyspora* species. JLB (IRb /LSC), JSB (IRb/SSC), JSA (SSC/IRa) and JLA (IRa/LSC) denote the junction sites between each corresponding region in the genome.

Figure 2 shows the expansion and contraction of the IR regions of 6 *Polyspora* species. During the evolution of cp genomes, there was little difference among the six *Polyspora* species. Both the length range of IR regions (26,077 bp-26,105 bp) and their junction with SC regions were highly conservative. The *rps19* gene of all *Polyspora* species were similar in location, 233 bp in the LSC region and 46 bp in the IRb region, while gene *rpl2* was entirely located in the IRb region. The *ndhF* gene of all species were located in the SSC region, 66 bp away from the IRb/SSC border in *P. axillaris*, *P. chrysandra* and *P. hainanensis*, while it was 10 bp shorter in *P. speciosa*, *P. longicarpa* and *P. tiantangensis*. The *ycf1* gene in all genomes spanned over the junction of the SSC and IRa regions. Compared to *P. longicarpa* and *P. tiantangensis*, the *ycf1* gene of the other 4 species were 6 bp longer in the SSC region, which led to the differences of gene length. Finally, the *trnH* gene in all Chinese *Polyspora* species were located on the IRa/LSC boundary line, and its distance from the junction was 1 bp.

### Long repeat sequence analysis

**Figure 3:**
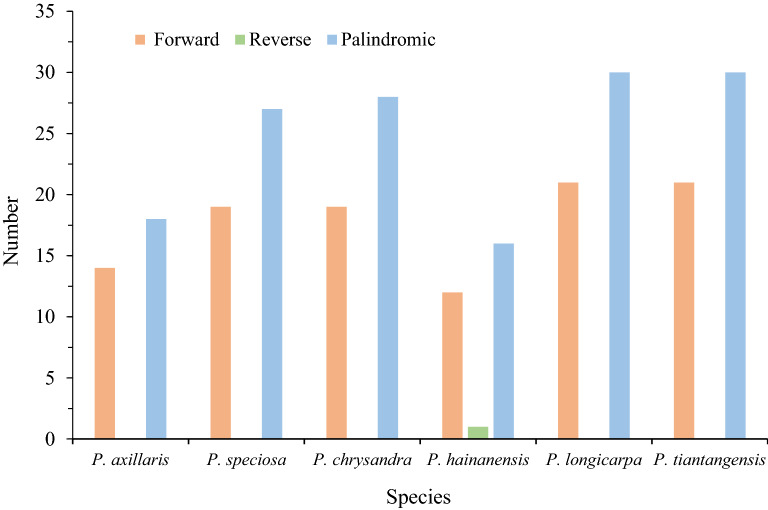
Number of long repeats in the cp genome sequence of Chinese *Polyspora* species.

**Figure 4:**
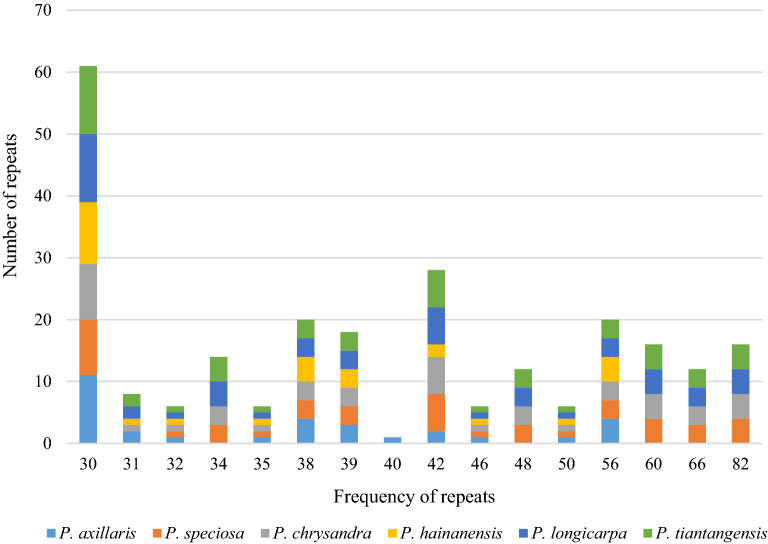
Frequency of the repeats more than 30 bp long.

A total of 256 long repeats were identified by using REPuter for six cp genomes (Table S3). Five species had only forward and palindromic repeats, and only *P. hainanensis* had reverse repeats. Complementary repeats did not exist in all Chinese *Polyspora* species (Fig. 3). Except for IR regions, the repeats length ranged from 30 to 82 bp, repeats length of 30, 42, 38 and 56 bp were accounted for the most common lengths (Fig. 4, Table S4). Among the six *Polyspora* species, *P. hainanensis* had the least number of repeats, including 12 forward, 16 palindromic and 1 reverse repeats (Fig. 3**,** Table S3). The number of forward repeats varied from 12 (*P. hainanensis*) to 21 (*P. longicarpa* and *P. tiantangensis*), and the number of palindromic repeats varied between 16 (*P. hainanensis*) and 30 (*P. longicarpa* and *P. tiantangensis*) (Fig. 3, Table S3). Most long repeats were located in IR regions (Table S4), while *ycf2* gene had the longest repeats at 82 bp.

### Simple sequence repeat (SSR) analysis

**Figure 5:**
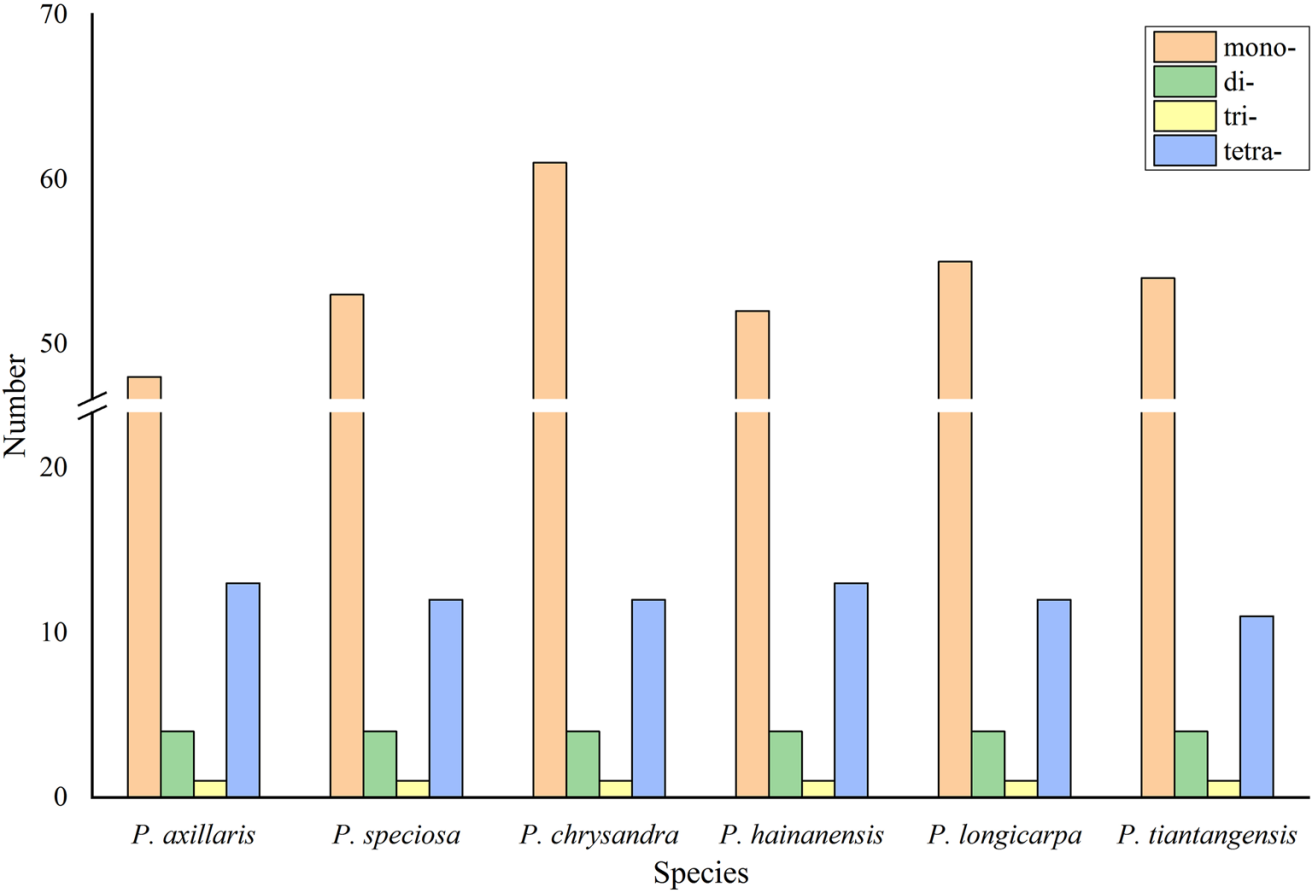
The comparison of SSRs distribution in the 6 *Polyspora* cp genomes.

**Figure 6:**
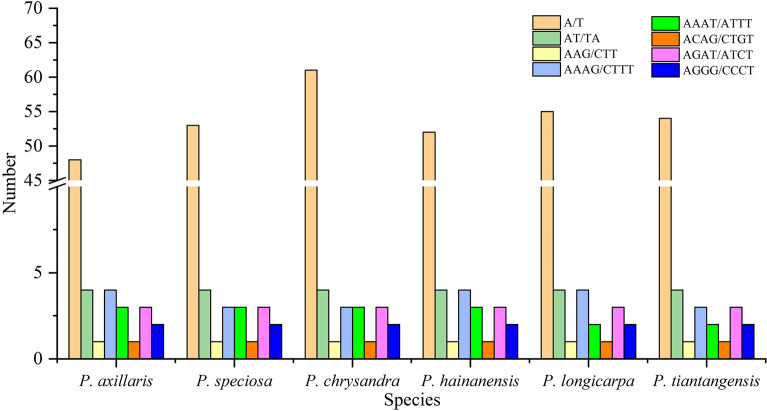
Number of different SSR types detected in the 6 *Polyspora* cp genomes.

The number of SSRs in the six *Polyspora* species ranged from 66 (*P. axillaris*) to 78 (*P. chrysandra*) (Table S5). Four kinds of SSRs were discovered, mononucleotide (mono-), dinucleotide (di-), trinucleotide (tri-) and tetranucleotide (tetra-). In each species of *Polyspora*, single nucleotide (mono-) repeats were the most common, and trinucleotide repeats accounted for the lowest proportion of SSRs (Fig. 5). The number of A/T mononucleotide repeats exceeded the total number of the other three types. AAG/CTT trinucleotide repeats and ACAG/CTGT tetranucleotide repeats were accounted as only one in each *Polyspora* species (Fig. 6, Table S6). The SSRs were mainly distributed in intergenic regions, few were found in coding regions: *psbI*, *atpA*, *rpoC2*, *rpoB*, *ycf3*, *atpB*, *rpoA*, *rpl16*, *rpl2*, *ccsA*, *ndhD*, *ndhA*, *ycf1* and *ycf2*. Among them, four genes (*ycf3*, *ndhD*, *ndhA*, *ycf1* ) were found to contain at least two SSRs, *ycf1* gene had the most SSRs.

### Comparative genomic analysis within Chinese Polyspora

**Figure 7:**
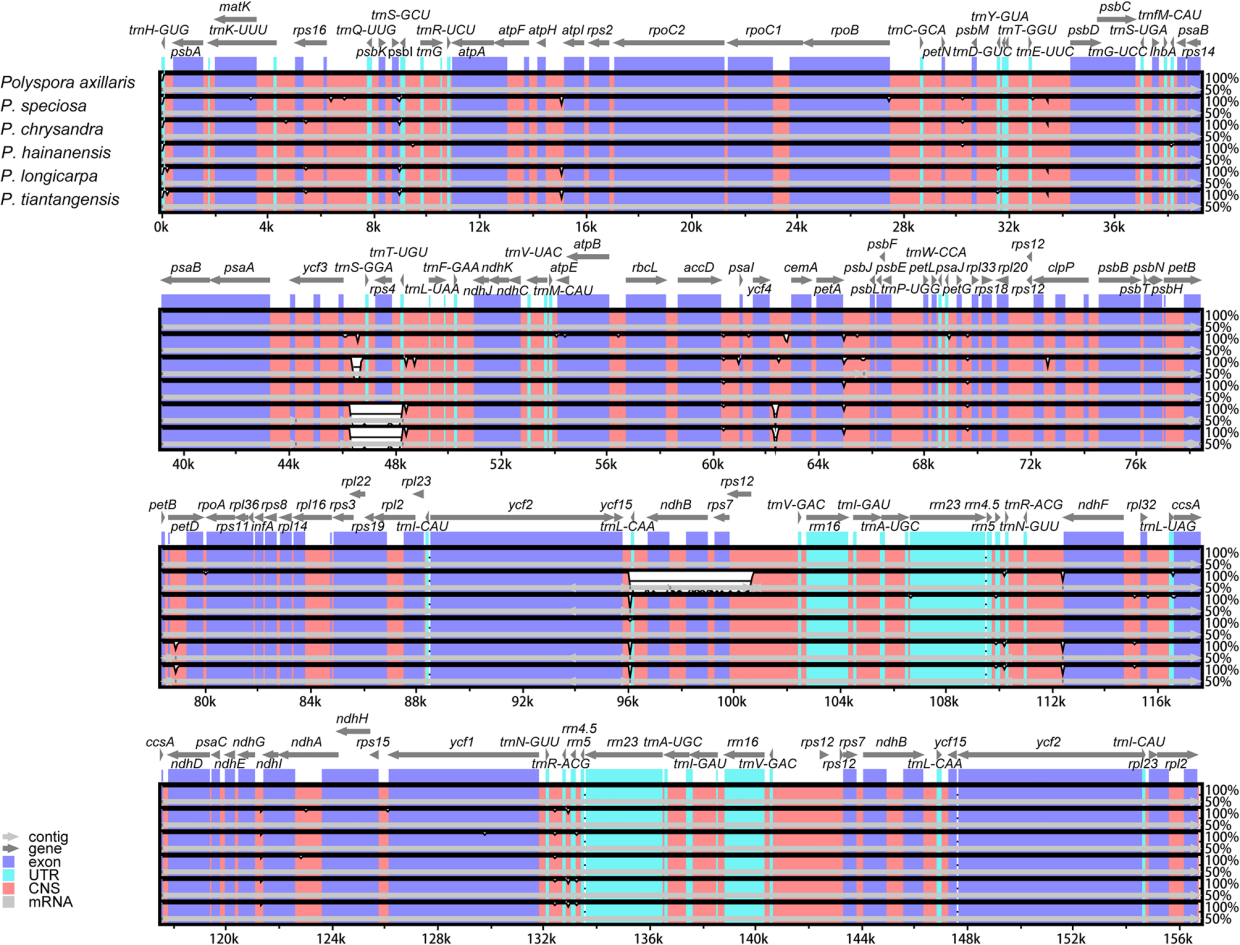
Alignment of Chinese *Polyspora* cp genomes in mVISTA.

To evaluate the differences of cp genomes among six *Polyspora* species, we performed mVISTA analysis with the annotated *P. axillaris* cp genome as a reference. The cp genomic sequences of the six species were relatively similar, and the sequence variation were mainly concentrated in the non-coding region, while the exons and untranslated regions (UTR) had little variation between genomes (Fig. 7). The coding regions were more conservative than the non-coding regions. Eleven intergenic spacer regions (*trnH-psbA*, *psbI-trnS*, *trnT-psbD*, *trnT-trnL*, *accD-psaI*, *ycf4-cemA*, *petA-psbJ*, *psaJ-rpl33*, *ycf15-trnL*, *ndhG-ndhI*, *trnN-trnR*) had the highest levels of divergence (Fig. 7). Mauve algorithm showed that no large fragments of gene rearrangement were found in the cp genome sequences of Chinese *Polyspora* species (Fig. S1).

### Divergence hotspots

**Figure 8:**
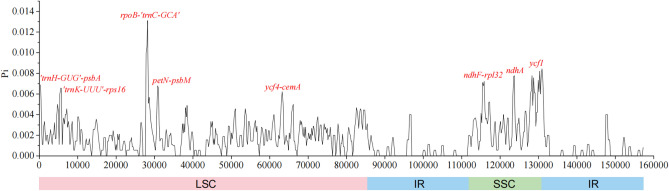
Nucleotide diversity values among six *Polyspora* species. X-axis, position of the midpoint of a window; Y-axis, nucleotide diversity of each window. (window length: 600 bp, step size: 200 bp).

The whole cp genome sequences of six *Polyspora* species were sliding from scratch by using DnaSP v6 software. Nucleotide diversity (Pi) values among the sequences were calculated and analyzed at 600 bp window length. There were 560 mutation sites in the aligned genome sequences, the Pi value ranged from 0.00056 to 0.01311, and the average value was 0.00227. Eight highly variable regions with Pi value greater than 0.005 were located precisely. These regions included *rpoB-trnC-GCA*, *ycf1*, *trnH-GUG-psbA*, *trnK-UUU-rps16*, *petN-psbM*, *ycf4-cemA*, *ndhF-rpl32* and *ndhA* (Fig. 8). Five of these gene segments were located in the LSC region, with the other three located in the SSC region. Two of the divergent regions (*ycf1* and *ndhA*) were presented at coding regions, others were in the non-coding regions. The LSC and SSC regions showed higher sequence divergence than the IR regions, and the protein-coding genes were more conserved than non-coding sequences. The *rpoB-trnC-GCA* region had the highest divergence value of 0.01311 (Fig. 8).

### Selective pressure analyses

**Figure 9:**
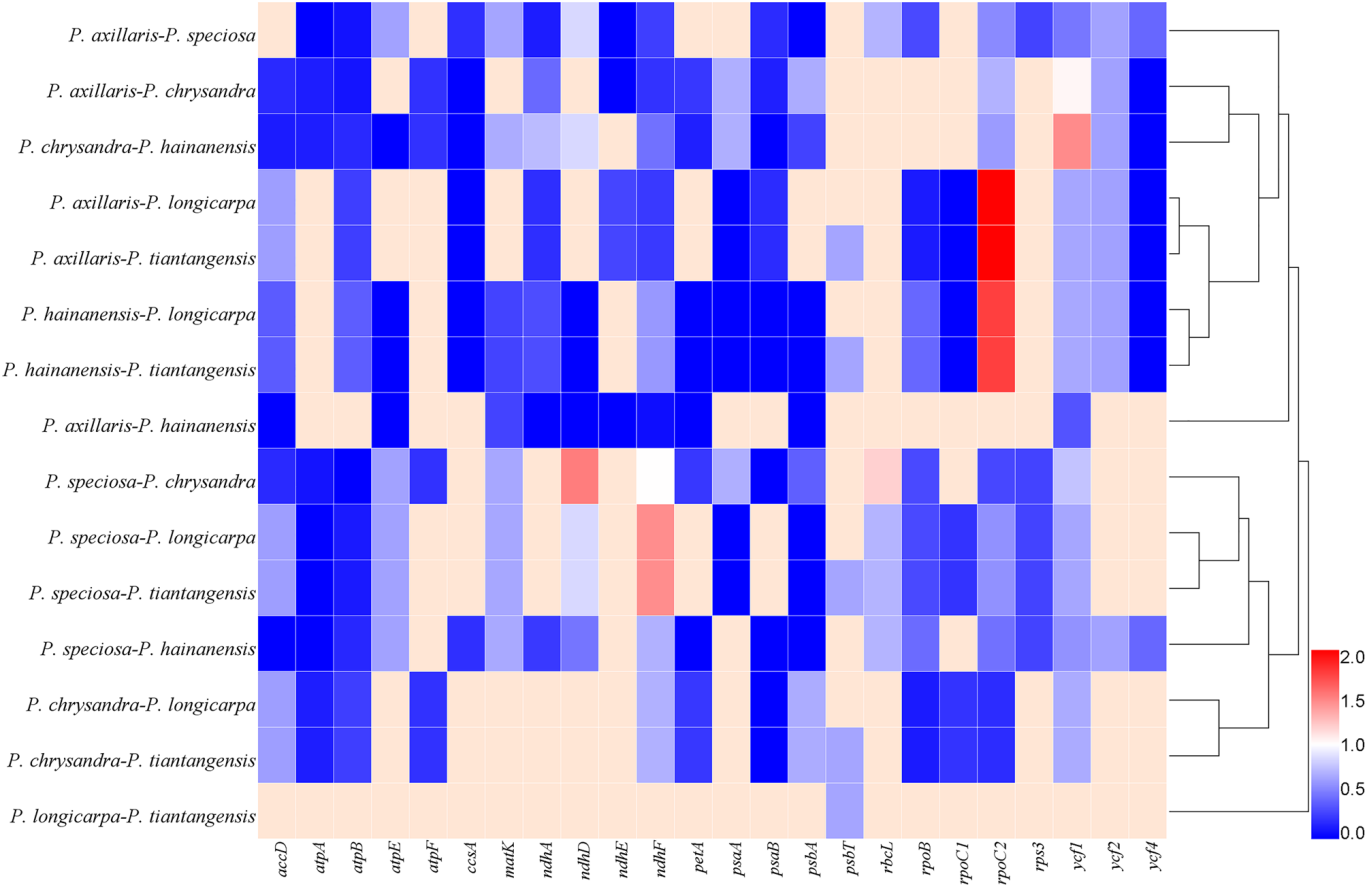
Pairwise Ka/Ks ratios of Chinese *Polyspora* species in different genes. The color closer to red represents the gene has a high Ka/Ks ratio.

We calculated the ratios of non-synonymous (Ka) and synonymous (Ks) substitutions by 80 protein-coding genes in *Polyspora*. The average Ka/Ks ratio for 80 protein genes analyzed in the six cp genomes was 0.3889 (Table S7). There were significant differences in the evolutionary rates among the six species, 24 of 80 protein genes had positive sites, of which 5 genes Ka/Ks > 1 (*ndhD*, *ndhF*, *rbcL*, *rpoC2*, *ycf1*) , suggesting that these five genes might be in the positive selection procedure of evolution (Fig. 9, Table S7). Especially, *rpoC2* gene had Ka/Ks ratios around 2.0 in four pairwise species (*P. axillaris*/*P. hainanensis* vs. *P. longicarpa*/*P. tiantangensis*), *rpoC2* was a candidate gene for adaptive evolution. The Ka/Ks ratios of most other genes were less than 0.5, indicating that overwhelming majority genes were undergoing strong purifying selection pressure.

**Figure 10:**
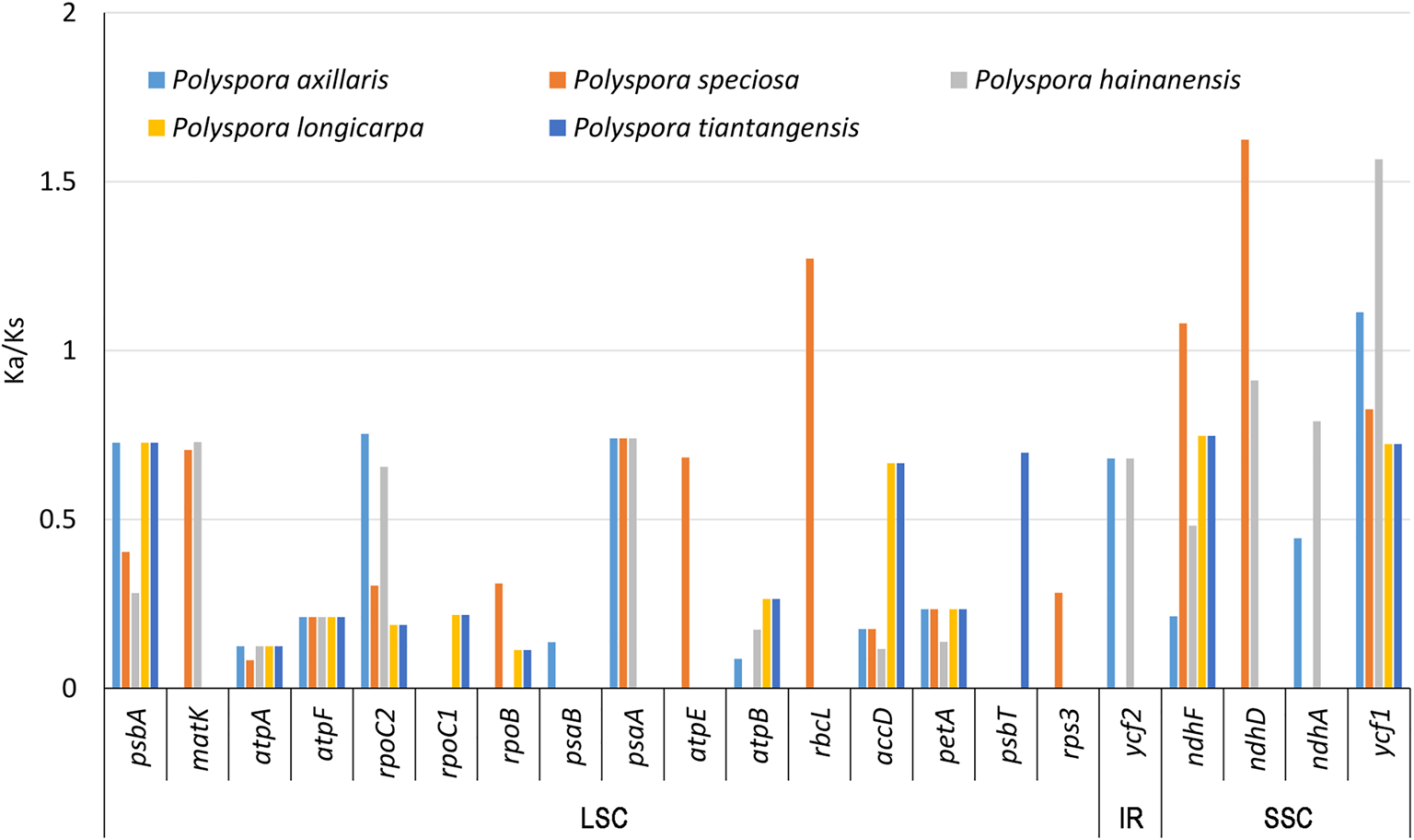
The Ka/Ks ratios of 21 protein-coding genes of *P. chrysandra* cp genome when compared to other five Chinese *Polyspora.*

We also analyzed Ka/Ks ratios of *P. chrysandra* cp genome versus other five Chinese *Polyspora* in detail (Fig. 10, Table S8). The result showed that 21 out of 80 protein-coding genes had positive sites, with the Ka/Ks ratios of three genes (*rbcL*, *ndhF*, *ndhD*) between *P. chrysandra* and *P. speciosa* being greater than 1. The *ycf1* gene in 2 pairs (*P. chrysandra* vs. *P. hainanensis*, *P. chrysandra* vs. *P. tiantangensis*) had Ka/Ks ratio > 1. Besides, the *psbA*, *matK*, *rpoC2*, *psaA*, *atpE*, *accD*, *psbT*, *ycf2* and *ndhA* genes, with Ka/Ks ratio > 0.5, were found in 17 comparison pairs. The Ka/Ks ratios in different regions (LSC, IR, SSC) were not regionally specific.

### Codon usage

**Figure 11:**
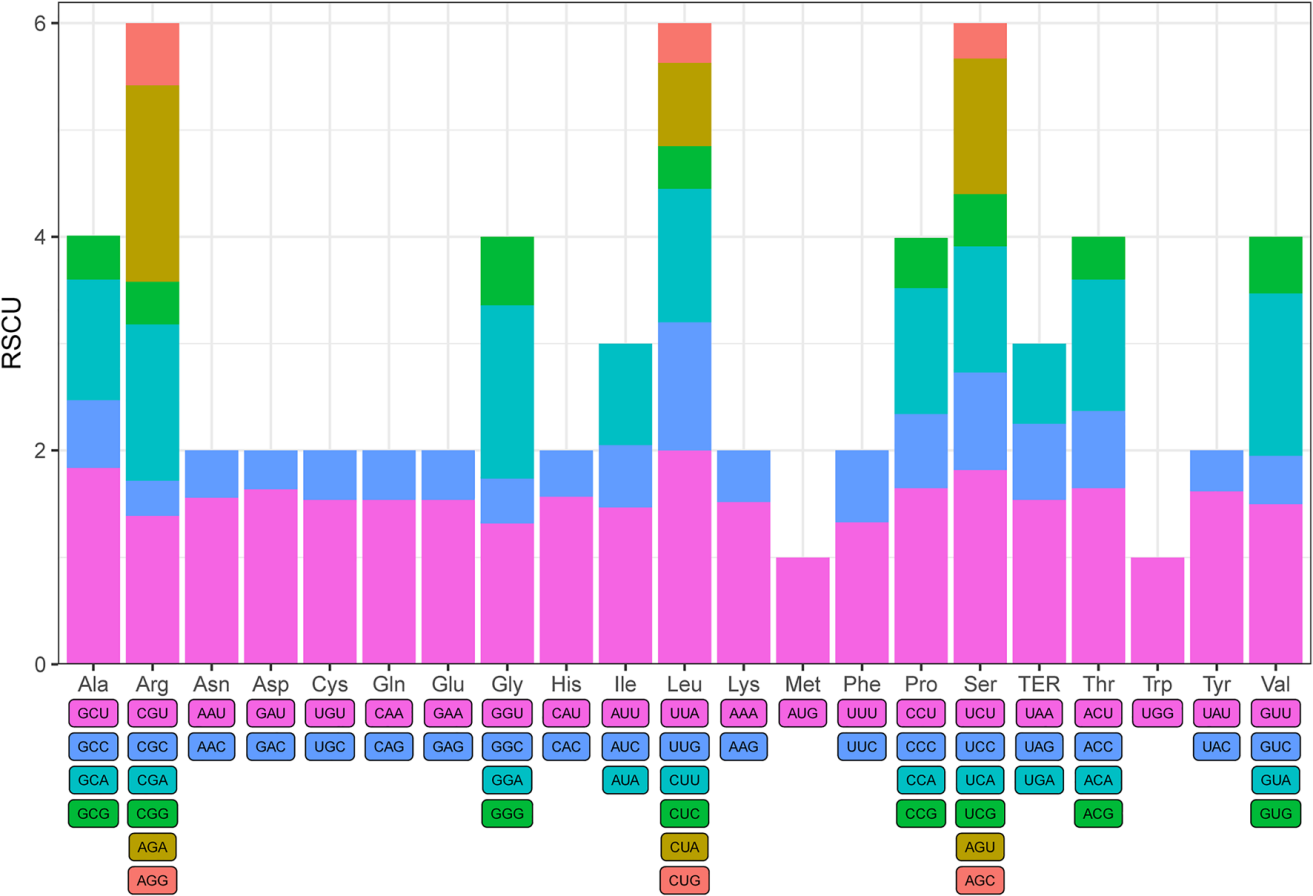
Codon content of 20 amino acids and stop codons in all protein-coding genes of *P. chrysandra* cp genome. Colors correspond to codons listed underneath the columns.

A total of 42 protein coding sequences were selected for codon usage analysis. The codon number and RSCU values of the six *Polyspora* species were similar, they were encoded 22,989 (*P. tiantangensis*) to 23,003 (*P. speciosa*) codons with the RSCU values ranging from 0.33 (CGC and AGC) to 2.00 (UUA) (Table S9). Among the protein-coding cp genes in the six *Polyspora* species, 20 amino acids were encoded by 64 types of codons. The codon usage bias of *P. chrysandra* was described in detail. Leucine with 2,386 codons (approximately 10.37% of the total) was the most abundant amino acid in the cp genome, cysteine with 250 codons (approximately 1.09% of the total) was found as a rare amino acid. Among the 64 codons, AUU-encoded isoleucine had the highest number of occurrences (986), and UGC-encoded cysteine had the lowest number of occurrences (57) (Table S9); AGC-encoded serine had the lowest RSCU values, and UUA-encoded leucine had the highest RSCU values. Tryptophan (UGG) and methionine (AUG) are encoded by only one codon without codon bias. There were 29 codons with RSCU values greater than 1, indicating biased usage (Fig. 11**,** excluding terminating codons). Among them, 16 ended with T, 12 ended with A, and only one ended with G. This indicates that the codon of *Polyspora* cp genome had a strong A/T preference in the third codon. This phenomenon is similar to the codon bias of cp genomes in most angiosperm.

### Phylogenetic analysis

**Figure 12:**
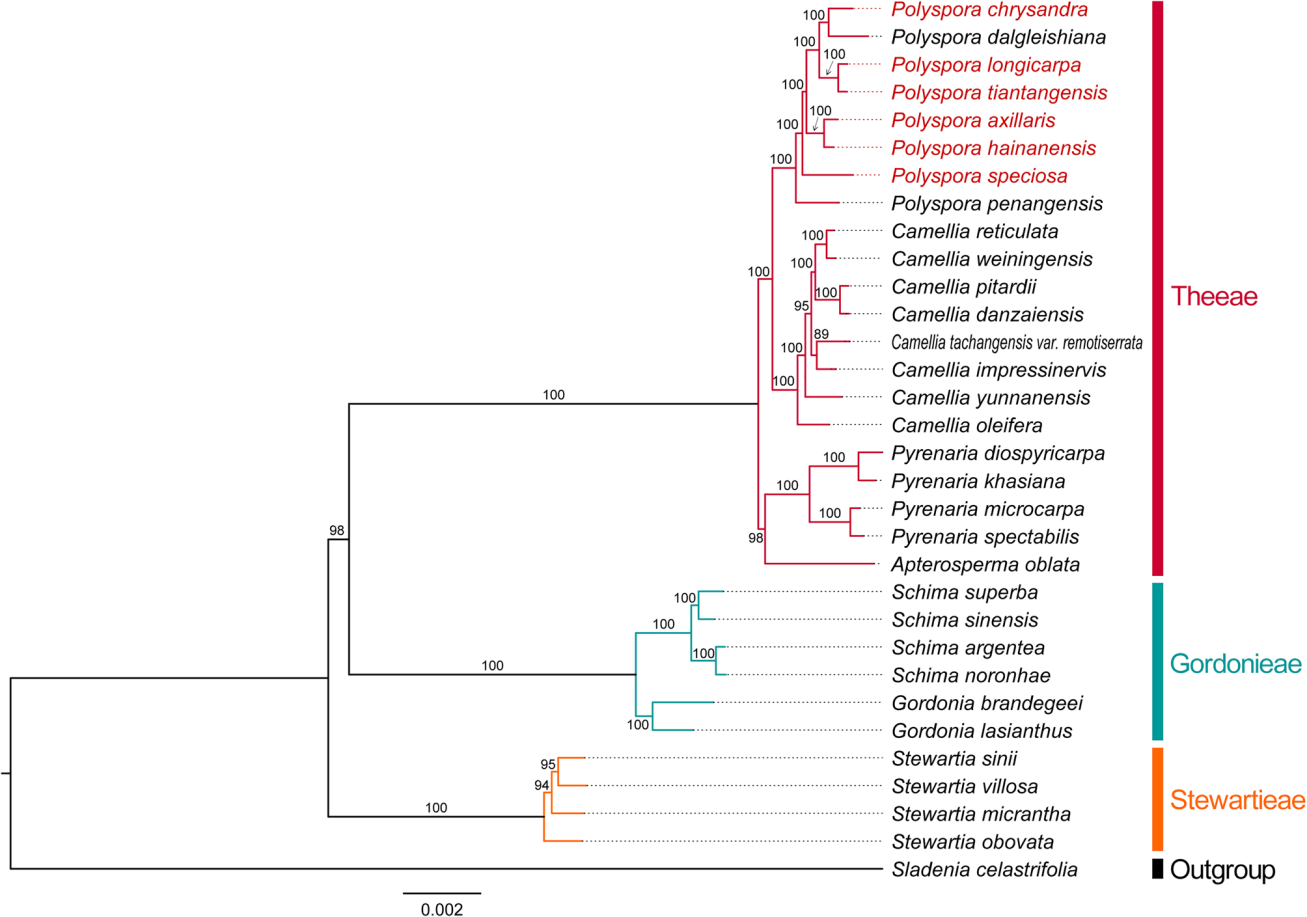
The maximum likelihood (ML) phylogenetic tree of the Theaceae family based on cp genome sequences. The red font of species name shows Chinese *Polyspora*.

We adopted 31 cp genomes from three tribes of Theaceae (including *P. chrysandra* cp genome newly sequenced in this study) to infer the phylogenetic relationships by using the maximum likelihood (ML) method. The ML tree generated 29 nodes, most of which had 100% bootstrap support. Except for one node with a bootstrap value of 89%, all other clades were supported with bootstrap values greater than 94% (Fig. 12). These 31 cp genome sequences were classified into three monophyletic groups, representing three tribes of Theaceae family. Four genera of *Polyspora*, *Camellia*, *Pyrenaria* and *Apterosperma* gathered into Theeae tribe. *Genus Gordonia*, which fruit morphological characteristics were similar to *Polyspora*, aggregated with *Schima* into tribe Gordonieae. Eight *Polyspora* species with published cp genome participated in phylogenetic analysis, six of them were from China, and the other two were from Vietnam (*P. dalgleishiana*) and Singapore (*P. penangensis*). These eight *Polyspora* species clustered into one group, with the bootstrap values of all internal nodes reached 100%. The eight *Polyspora* species were split into two groups, *P*. *penangensis*, which is native to Peninsular Malaysia and Singapore, formed sister groups with the other seven species. *P. chrysandra* was most closely related to *P. dalgleishiana* from Vietnam. Together, the three groups of Theaceae based on the cp genomes were consistent with the traditional taxonomy.

## Discussion

In this study, we characterized the complete cp genome of *P. chrysandra*, and compared with other five Chinese *Polyspora* species. We found that the cp genome size, gene content, structure and other characteristics were highly conserved among the six *Polyspora* species, and no gene rearrangement was detected **(**Fig. S1**)**. The cp genome size of most angiosperms range from 120 to 160 kb, GC contents range from 30 to 40%, and IR region length range from 20 to 28 kb^25^. The cp genome lengths of the six *Polyspora* species from China were 156,452–157,066 bp, the IR region lengths were 26,007–26,105 bp, and the GC content was 37.3% (Table S2), which were consistent with the characteristics of the cp genomes of angiosperms. The GC content of Chinese *Polyspora* was similar to that of *Camellia* species published by Huang et al^26^ and Li et al^27^. The variation of cp genome size ranged from 1 to 614 bp, *P. chrysandra* was greatly differed from other species, while the difference of cp genome length between *P. tiantangensis* and *P. longicarpa* was only 1 bp. Although plant cp genomes are conservative in size and structure, IR expansion/contraction are common evolutionary phenomenon^26^. Through the comparative analysis of the IR boundary regions, we can clearly see the differences of chloroplast genomes among different species of *Polyspora*. Compared with the other five species of Chinese *Polyspora*, *P. chrysandra* had the shortest cp genome length (156,452 bp), but the IR region (26,105 bp) was the longest among the six species, indicating that an expansion existed. Based on the *ndhF* gene location in IRb/SSC region, the six species can be divided into two groups, group A (*P. axillaris*, *P. chrysandra*, *P. hainanensis*) and group B (*P. speciosa*, *P. longicarpa*, *P. tiantangensis*) (Fig. 2), which is consistent with the morphological taxonomy. The three species in group A are shrubs or small trees, while those in group B are large trees. The length of *ycf1* gene located in SSC/IRa boundary could also be used to distinguish *P. speciosa* from *P. longicarpa* and *P. tiantangensis* in group B, where the *ycf1* gene of *P. speciosa* was 6 bp longer than that of the other two species. However, the contraction and expansion of IRs in group A were identical. The phenomenon of IR expansion and the features of genome boundaries could provide useful information for *Polyspora* taxonomy.

Repeats sequences play an important role in genome rearrangement, recombination and inversion^28–31^. Furthermore, repetitive sequences are important inducements of base indels and substitutions in cp genome^29,30^. In the long repeats of Chinese *Polyspora*, palindromic repeats were the most abundant, followed by forward repeats, and only *P. hainanensis* had reverse repeats. Palindromic repeats are involved in important life activities, such as DNA replication and transcriptional termination. *P. hainanensis* had the least number of repeats, and the number of long repeats of *P. longicarpa* and *P. tiantangensis* were exactly the same. SSRs are repeating sequences of typically 1–6 bp^29^, which are widely distributed in eukaryotic genomes^32^. SSRs of cp genome are usually used for population genetics and phylogenetic analysis^27,30^. A total of 66 ~ 78 polymorphic SSRs were identified in this study (Table S5), and these numbers are close to that of *Camellia* species^27^. Most SSRs were distributed in intergenic regions, whereas few in protein-coding genes, which were consistent with previous studies on angiosperm cp genome^30,33,34^. The composition of SSRs in the six *Polyspora* cp genomes were similar to that of most angiosperms, with A/T single nucleotide (mono-) repeats dominated all repeat units, which might be one of the reasons for the abundance of A/T bases in the cp genomes. The long repeats and SSRs identified in this study can provide effective information for the detection of interspecific and intraspecific polymorphism of *Polyspora*, and can be used as molecular markers to evaluate the genetic diversity of wild populations of Theaceae species.

Due to the highly conservative structure of plant cp genomes, mutational hotspots can be easily identified by comparative analyses^21^. These mutation hotspots surrounded by conserved sequences are commonly used as DNA barcodes in population genetic or phylogenetic studies^21,35^. According to the comprehensive analysis of sequence differences in mVISTA and nucleotide variation inferred by DnaSP, we found that the sequence variation of the genus *Polyspora* mainly occurred in the non-coding region and intergenic region. In the analysis of cp genome sequence variation, we detected eleven divergent regions (*trnH-psbA*, *psbI-trnS*, *trnT-psbD*, *trnT-trnL*, *accD-psaI*, *ycf4-cemA*, *petA-psbJ*, *psaJ-rpl33*, *ycf15-trnL*, *ndhG-ndhI*, *trnN-trnR*) (Fig. 7). Based on our sliding window analysis, *rpoB-trnC-GCA, ycf1, trnH-GUG-psbA, trnK-UUU-rps16, petN-psbM, ycf4-cemA, ndhF-rpl32 and ndhA* were highly variable (Fig. 8). In fact, *trnH-psbA* and *ycf1* have been used as standard barcodes in most plants, and *ycf1* have been considered as the most promising plastid DNA barcode in terrestrial plants^36^. Further research is necessary to investigate which of these high variation genes or gene spacers could be used as reliable and effective DNA barcodes in genus *Polyspora*.

Adaptive evolution analysis of cp genome is an important part of studying gene function and gene structure changes^37^. In the process of cp genome evolution, most genes are selected for purification, some genes are involved in environmental adaptation and positive selection, while others are in neutral evolution^38^. We identified 24 genes with positive loci in six *Polyspora* species, five of them (*ndhD*, *ndhF*, *rbcL*, *rpoC2*, *ycf1*) with Ka/Ks values greater than 1 (Fig. 9, Table S7), these genes may have been undergoing certain functional diversification during their evolve adaptation. These genes were from three different functional groups, including photosystems (*ndhD*, *ndhF*, *rbcL*), expression-related genes (*rpoC2*), and unclassified genes (*ycf1*) (Table 1). The *ndh* gene encodes cp NADH dehydrogenase^39^. The NADH dehydrogenase complex of higher plants are not only involved in photosynthetic electron transport^40^, but also resistant to photooxidative stress^41^. For example, *ndhF* shows positive selection effect on Australia *Citrus* adaptation to dry and hot climates^42^. The *rbcL* gene encodes a large sub-unit of ribulose bisphosphate carboxylase, which plays an important role in catalyzing the fixation of CO_2_^43^. The *rpoC2* gene encodes the β" subunit of cp RNA polymerase^39^, *rpoC2* gene had Ka/Ks ratios around 2.0 in four *Polyspora* pairwise species, which might be related to the function of their cp RNA polymerase. Gene *ycf1* is one of the largest open reading frames in cp genome^39^. Although its specific function is still unclear, it has low sequence similarity among different species and is used as one of the commonly DNA barcodes. The Ka/Ks values of *ycf1* gene varies greatly among different phytogroup^44^, and the evolutionary significance of this gene needs to be further studied. In conclusion, these positive selection genes may contribute to the diversification and adaptive evolution of *Polyspora*.

Codon is the key to the accurate expression of genetic information^14^. Codon usage bias is a common phenomenon in plants. It is generally considered to be an intricate combined outcome of natural selection, mutation, and genetic drift during the long-term evolution of species and genes^45^, which reflects the different pressures of different genes or genomes in the course of evolution^32^. The total number of codons in six *Polyspora* were different, which were 22,993 in *P. axillaris* and *P. hainanensis*, 23,003 in *P. speciosa*, 22,999 in *P. chrysandra*, 22,997 in *P. longicarpa* and 22,989 in *P. tiantangensis* (Table S9). This might have been caused by the different cp genome sizes among different species. Many previous studies have shown that leucine and isoleucine codons are the most common codons in chloroplasts, while cysteine codons are the rarest^28,30,46^. The six *Polyspora* species in this study also accord with this feature. In addition, taking *P. chrysandra* as an example, it was found that most of the codons with RSCU value greater than 1 ended with A/T base, whereas most of the codons with RSCU value less than 1 ended with C/G base (Table S9), which might be due to the abundant content of A/T in the cp genome of *Polyspora*. Similar phenomenon has been reported in *Michelia shiluensis*^47^, *Nephelium lappaceum*^34^ and many other plants. The study of codon preferences can help us better understand the gene expression and molecular evolution mechanisms of the genus *Polyspora*.

Theaceae is an important components of subtropical evergreen broad-leaved forest in China^48^. Since the twenty-first century, Prince & Parks^49^ have revealed the phylogenetic framework of Theaceae by using chloroplast-encoded *rbc*L and *mat*K + flanking intergenic spacer region data, and divided Theaceae into three tribes, namely Theeae, Gordonieae, and Stewartieae. Yu et al.^50^ used complete plastid genome and nuclear ribosomal DNA data to better solve the phylogenetic relationships among the three tribes in Theaceae. The molecular phylogenetic relationships of the three tribes are consistent with their endosperm evolution model. Tribe Stewartieae has rich endosperm, tribe Gordonieae contains a thin layer of endosperm and tribe Theeae has no endosperm^51^. A number of subsequent phylogenetic studies related to Theaceae have reached similar conclusions^1,5,24,52^. However, due to incomplete sequencing data, none of these studies included all the Chinese *Polyspora* species. In this study, we used complete cp genome sequences to reconfirm the three branches of Theaceae. At the same time, we revealed the phylogeny of Chinese *Polyspora* species and their phylogenetic location within Theaceae (Fig. 12). Among the phylogenetic relationships of the six *Polyspora* species, *P. speciosa* is relatively independent. *P. speciosa* has the earliest differentiation, the widest distribution and the most abundant variation in China. *P. chrysandra* is most closely related to *P. dalgleishiana* from Vietnam, the main distribution area of *P. chrysandra* is Yunnan Province of China, which is adjacent to Vietnam, and the two species are relatively lately differentiated. *P. axillaris* has the closest relationship with *P. hainanensis*, which is also consistent with their geographical distribution. *P. axillaris* is mainly distributed in Guangdong Province of China, while *P. hainanensis* is only distributed in Hainan Province of China, and the two provinces are separated by Qiongzhou Strait. Strait isolation may be responsible for the lineage differentiation of the two species. *P. tiantangensis* and *P. longicarpa* are grouped together, which not only have the same geographical distribution, but also have very similar morphological characteristics. The differences are that the leaf apex of *P. tiantangensis* is slightly concave, and the capsule is 6–8 locular^53^. The cp genome lengths of the two species are only 1 bp different, the IR and SC boundary genes locations, the number of repeat sequences, and the sequence variation features are all the same, which implies that they may be the same species. However, due to hybridization and polyploidization, the cp and nuclear genomes evolved independently, and the phylogeny of cp genomes alone is insufficient in making taxonomic decisions^1,54^. In addition, the genus *Polyspora* includes nearly 50 species, mainly distributed in South and Southeast Asia. Only six *Polyspora* species from China were studied in this study. Therefore, further studied with more species are needed to help better understand the phylogenetic relationship of *Polyspora*.

## Conclusion

The complete cp genome of *P. chrysandra* was sequenced and reported for the first time in this study. Subsequently, comparative genomic analyses were performed with other Chinese *Polyspora* cp genomes. The cp genomes of six species in the genus *Polyspora* from China were typical tetrad structures, and the GC content was 37.3%. The cp genome structure, segment length and gene content of all species were highly conserved. In addition, abundant long repeats sequences, SSRs and highly variable regions were identified, which can provide rich genetic information for the study of genus *Polyspora*. In particular, eight highly variable gene regions (*rpoB-trnC*, *ycf1*, *trnH-psbA*, *trnK-rps16*, *petN-psbM*, *ycf4-cemA*, *ndhF-rpl32* and *ndhA*) were identified with good potential as DNA barcodes for Phylogeography and genetic diversity studies in *Polyspora*. Five genes (*ndhD*, *ndhF*, *rbcL*, *rpoC2* and *ycf1*) were detected with Ka/Ks ratio greater than 1, suggesting that these genes have undergone positive selection during evolutionary adaptation. Studies of codon usage showed that some amino acids had a pronounced bias, which may assist us in the understanding of the gene expression and molecular evolution mechanism of *Polyspora*. Phylogenetic tree based on the complete cp genomes revealed the phylogenetic relationships among *Polyspora* species, providing an in depth understanding of the phylogenetic relationships of the three tribes of Theaceae. In conclusion, our results may provide insights into the evolution and phylogeny of cp genome of genus *Polyspora* and even Theaceae.

## Methods

### Data collection, DNA extraction and sequencing

Fresh leaves of *Polyspora chrysandra* were collected from Shiping county of Yunnan province, China (23°54′15″N, 102°27′17″E). A voucher specimen (FC20201015) was identified and deposited in the Herbarium of Southwest Forestry University (SWFU) by Chang-Le Ma & Zhi-Feng Fan. Total genomic DNA was extracted using CTAB method^55^ and sequenced with the Illumina NovaSeq 6000 platform at the Novogene Company (Beijing, China). High-quality clean reads were obtained from filtered raw reads using NGS QC Toolkit version 2.3.3 with default parameters^56^. Other five Chinese *Polyspora* cp genomes were downloaded from NCBI Genbank, including *P. axillaris* (NC_035645^50^), *P. speciosa* (NC_035643^50^), *P. hainanensis* (NC_035693^50^), *P. longicarpa* (NC_035689^50^) and *P. tiantangensis* (NC_053889^5^).

### Chloroplast genome assembly and annotation

The chloroplast genome was de novo assembled by GetOrganelle^57^. Gene annotation was performed by Geneious R11 (https://www.geneious.com) with the *P. axillaris* (NC_035645) cp genome sequence as a reference. The circular chloroplast genome map was drawn by OGDRAW version 1.3.1^58^. Finally, the annotated sequence was submitted to GenBank (accession number MW801387).

### Repeat structure identification

REPuter was used to detect four kinds of long repeats: forward, reverse, palindromic, and complementary repeats^59^. Detection parameter settings were used as follows: repfind –c -f –p -r -l 30 -h 3 -best 1000. Simple sequence repeats (SSR) identification was carried out by MISA^60^, and the minimum thresholds for mono-, di-, tri-, tetra-, penta-, and hexa-nucleotides were set to 10, 5, 4, 3, 3, and 3, respectively.

### Sequence variation of chloroplast genome

Comparisons between *Polyspora chrysandra* and other five Chinese *Polyspora* cp genomes were visualized using mVISTA^61^ with the annotation of *P. axillaris* as the reference in shuffle-LAGAN mode^62^. The borders of four different regions among the six cp genomes of *Polyspora* were visualized using IRscope^63^. The genome rearrangement of the genus was detected by Mauve algorithm^64^. The multiple sequence alignment for cp genome was conducted using MAFFT^65^. For investigating the nucleotide diversity (Pi), coding and non-coding regions were extracted and computed with DnaSP v6^66^, window length was set to 600 bp, and the step size was set as 200 bp.

### Selective pressure analyses

The 80 protein-coding genes of six Chinese *Polyspora* species were used to evaluate evolutionary rate variation. KaKs_Calculator2.0^67^ with MLWL model^68^ was used to determine the ratio of non-synonymous substitutions (Ka) and synonymous substitutions (Ks). Genes with Ka/Ks < 1 (especially less than 0.5) were considered as under purifying selection; genes with Ka/Ks > 1 indicates probable positive selection; genes with Ka/Ks values close to 1 indicate neutral evolution. When Ks = 0, the value of Ka/Ks was represented by NA, indicates that the gene has few nonsynonymous sites/substitutions, and was not considered in our analysis. The fas-clustal format conversion was implemented by ALTER^69^. The heatmap among each individual gene in the *Polyspora* species was drawn by HemI^70^.

### Codon usage

Since the effective codon number cannot be correctly calculated for short sequences^71^, in order to reduce sample error, we removed all coding sequences (CDS) shorter than 300 bp for identifying codon usage patterns. Finally, 42 of 80 CDSs were used for codon usage analysis. CodonW v.1.4.2 was used to obtain the relative synonymous codon usage (RSCU)^72^, the results were mapped by R software. RSCU is the ratio of the frequency of a specific codon to the expected frequency. RSCU > 1 means that the codon is used more frequently than expected, RSCU < 1 means that the codon is used less frequently than expected.

### Phylogenetic analysis

To construct the phylogenetic relationships and examine the phylogenetic status of Chinese *Polyspora* within Theaceae, the complete cp genomes of 30 Theaceae species were download from GenBank (Table S1), *Sladenia celastrifolia* (Sladeniaceae, NC_035707) was used as an outgroup. We aligned the 32 complete cp genomes (including *P. chrysandra*) with MAFFT online^73^, and removed the ambiguously aligned fragments using Gblocks^74^ algorithm in PhyloSuite v1.2.2^75^, with the following parameter settings: minimum number of sequences for a conserved/flank position (17/17), maximum number of contiguous non-conserved positions (8), minimum length of a block (10), allowed gap positions (with half). The nucleotide replacement saturation was verified by DAMBE6^76^. The best fitting nucleotide substitution model (GTR + I + G) was chosen by ModelGenerator v0.85^77^. Finally, the Maximum likelihood (ML) tree with 1000 bootstrap replicates was constructed by using RAxMLv8.2.12^78^ on the CIPRES Science Gateway platforms^79^. Phylograms were visualized with FigTree v1.4.4 (http://tree.bio.ed.ac.uk/) and decorated in Adobe Illustrator.

### Ethics statement

The plant materials used in this study were obtained from wild plants and did not involve any endangered or protected species. The materials were collected under the permission of the Forestry and Grassland Bureau of Shiping County, Yunnan Province, China. The plant materials were identified by Chang-Le Ma and Zhi-Feng Fan.

### Plant guideline statement

Experimental research and field studies on plants, including the collection of plant material, complied with relevant institutional, national, and international guidelines and legislation.

## Supplementary Information

Supplementary Information 1.

Supplementary Information 2.

Supplementary Information 3.

Supplementary Information 4.

## Data Availability

The cp genome sequence of *P. chrysandra* was submitted on the National Center for Biotechnology Information (NCBI), and the accession number was MW801387 (https://www.ncbi.nlm.nih.gov/nuccore/MW801387).

## References

[1] Choo, L. M., Niissalo, M. A., Leong, P. K. F. & Khew, G. S. The complete plastome sequence of *Gordonia penangensis* Ridl. supports the transfer of Asian *Gordonia* into *Polyspora* (Theaceae). *Phytotaxa* **458**, 159–166 (2020).

[2] Nguyet HNL (2020). An updated checklist of Theaceae and a new species of *Polyspora* from Vietnam. Taiwania.

[3] Ma CL, Li J, Bai Q, Huang XX, Cheng XM (2015). Distribution and utilization of the indigenous tree species of genus *Polyspora* in Yunnan province. Heilongjiang Agric. Sci..

[4] Fan ZF, Han L, Ma CL (2021). Research advances of *Polyspora* Sweet (Theaceae). Guihaia.

[5] Fan ZF, Qian SJ, Zhang YH, Ma CL (2021). Characterization of the complete chloroplast genome of *Polyspora tiantangensis* (Theaceae), an endemic and endangered species in southwestern China. Mitochondrial DNA B Resour..

[6] Li Y (2017). Microwave-assisted extraction of natural antioxidants from the exotic *Gordonia axillaris* fruit:optimization and identification of phenolic compounds. Molecules.

[7] Li Y (2019). Polyphenolic profile and antioxidant capacity of extracts from *Gordonia axillaris* fruits. Antioxidants.

[8] Fu, H. Z. *Studies on the chemical constituents and bioactivities of Gordonia chrysandra and Gordonia kwangsiensis*, Chinese Academy of Medical Sciences & Peking Union Medical College (2012).

[9] Tang, J. *Studies on chemical constituents and bioactivities of Polyspora longicarpa: synthesis and bioactivities of carbazole alkaloid Claulansine F*, Chinese Academy of Medical Sciences & Peking Union Medical College (2013).

[10] Xu L, Ren Q, Wan KH, Fu HZ (2019). Chemical constituents from stem of *Gordonia Chrysandra*. J. Chin. Med. Mater..

[11] Yang SX (2004). Reassessing the relationships between *Gordonia* and *Polyspora* (Theaceae) based on the combined analyses of molecular data from the nuclear, plastid and mitochondrial genomes. Plant Syst. Evol..

[12] Raman G, Park S (2016). The complete chloroplast genome sequence of *Ampelopsis*: gene organization, comparative analysis, and phylogenetic relationships to other angiosperms. Front. Plant Sci..

[13] Dong W (2016). Comparative analysis of the complete chloroplast genome sequences in psammophytic *Haloxylon* species (Amaranthaceae). PeerJ.

[14] Zhou XJ (2020). Comparative analysis of chloroplast genome characteristics between *Paeonia jishanensis* and other five species of *Paeonia*. Sci. Silvae Sin..

[15] Asaf S (2017). Chloroplast genomes of Arabidopsis halleri ssp gemmifera and Arabidopsis lyrata ssp. petraea: structures and comparative analysis. Sci. Rep..

[16] Gu C, Ma L, Wu Z, Chen K, Wang Y (2019). Comparative analyses of chloroplast genomes from 22 Lythraceae species: inferences for phylogenetic relationships and genome evolution within Myrtales. BMC Plant Biol..

[17] Zheng G (2020). Comparative analyses of chloroplast genomes from 13 *Lagerstroemia* (Lythraceae) species: identification of highly divergent regions and inference of phylogenetic relationships. Plant Mol. Biol..

[18] Daniell H, Lin CS, Yu M, Chang WJ (2016). Chloroplast genomes: diversity, evolution, and applications in genetic engineering. Genome Biol..

[19] Dong W, Xu C, Cheng T, Lin K, Zhou S (2013). Sequencing angiosperm plastid genomes made easy: a complete set of universal primers and a case study on the phylogeny of Saxifragales. Genome Biol. Evol..

[20] Li Q, Guo QQ, Gao C, Li HE (2020). Characterization of complete chloroplast genome of *Camellia weiningensis* in Weining. Acta Hort. Sin..

[21] Hong Z (2020). Comparative analyses of five complete chloroplast genomes from the genus *Pterocarpus* (Fabacaeae). Int. J. Mol. Sci..

[22] Zhao YM (2019). Chloroplast genome structural characteristics and phylogenetic relationships of Oleaceae. Chin. Bull. Bot..

[23] Gunathilake, L. A. A. H. *Evolution of Polyspora ( = Gordonia;Theaceae) in Sri Lanka*, University of Miami (2015).

[24] Yu XQ (2020). The complete chloroplast genome sequence of *Laplacea alpestris* and its phylogenetic position. Mitochondrial DNA B Resour..

[25] Zhang T (2012). The complete chloroplast and mitochondrial genome sequences of *Boea hygrometrica*: insights into the evolution of plant organellar genomes. PLoS ONE.

[26] Huang H, Shi C, Liu Y, Mao SY, Gao LZ (2014). Thirteen *Camellia* chloroplast genome sequences determined by high-throughput sequencing: genome structure and phylogenetic relationships. BMC Evol. Biol..

[27] Li W, Zhang C, Guo X, Liu Q, Wang K (2019). Complete chloroplast genome of *Camellia japonica* genome structures, comparative and phylogenetic analysis. PLoS ONE.

[28] Asaf S (2017). The complete chloroplast genome of wild rice (*Oryza minuta*) and its comparison to related species. Front. Plant. Sci..

[29] Asaf, S. *et al.* Complete chloroplast genome sequence and comparative analysis of loblolly pine (*Pinus taeda* L.) with related species. *PLoS One* **13**, e0192966 (2018). 10.1371/journal.pone.0192966 PMC5875761 29596414

[30] Song W (2022). Comparative analysis the complete chloroplast genomes of nine *Musa* species: genomic features, comparative analysis, and phylogenetic implications. Front. Plant. Sci..

[31] Song W (2022). Comparative chloroplast genome analysis of wax gourd (*Benincasa hispida*) with three benincaseae species, revealing evolutionary dynamic patterns and phylogenetic implications. Genes.

[32] Liu K (2021). Comparative and phylogenetic analysis of complete chloroplast genomes in Eragrostideae (Chloridoideae, Poaceae). Plants.

[33] Cheng H (2017). The complete chloroplast genome sequence of strawberry (Fragaria x ananassa Duch.) and comparison with related species of Rosaceae. PeerJ.

[34] Dong F, Lin Z, Lin J, Ming R, Zhang W (2021). Chloroplast genome of Rambutan and comparative analyses in Sapindaceae. Plants.

[35] Abdullah (2020). Chloroplast genome of *Hibiscus rosa-sinensis* (Malvaceae): comparative analyses and identification of mutational hotspots. Genomics.

[36] Dong W (2015). ycf1, the most promising plastid DNA barcode of land plants. Sci. Rep..

[37] Nei M, Kumar S (2000). Molecular evolution and phylogenetics.

[38] Li P (2020). Comparison of the complete plastomes and the phylogenetic analysis of *Paulownia* species. Sci. Rep..

[39] Wang B, Gao L, Su YJ, Wang T (2012). Adaptive evolutionary analysis of chloroplast genes in euphyllophytes based on complete chloroplast genome sequences. Acta Sci. Natur. Univ. Sunyatseni.

[40] Joët T, Cournac L, Horvath EM, Medgyesy P, Peltier G (2001). Increased sensitivity of photosynthesis to antimycin A induced by inactivation of the chloroplast ndhB gene. Evidence for a participation of the NADH-dehydrogenase complex to cyclic electron flow around photosystem I. Plant Physiol..

[41] Horváth EM (2000). Targeted inactivation of the plastid ndhB gene intobacco results in an enhanced sensitvity of photosynthsis to moderate stomatal closure. Physiol. Plant..

[42] Caspermeyer J (2015). Into thin air and back: deer mouse study examines high-altitude adaptation. Mol. Biol. Evol..

[43] Azarin K, Usatov A, Makarenko M, Khachumov V, Gavrilova V (2021). Comparative analysis of chloroplast genomes of seven perennial *Helianthus* species. Gene.

[44] Shi C (2020). Complete chloroplast genomes of 14 Mangroves: phylogenetic and comparative genomic analyses. Biomed Res. Int..

[45] Ma LN, Cui P, Zhu J, Zhang ZH, Zhang Z (2014). Translational selection in human: more pronounced in housekeeping genes. Biol. Direct.

[46] Shahzadi I (2020). Chloroplast genome sequences of *Artemisia maritima* and *Artemisia absinthium*: Comparative analyses, mutational hotspots in genus Artemisia and phylogeny in family Asteraceae. Genomics.

[47] Deng Y (2020). Complete chloroplast genome of *Michelia shiluensis* and a comparative analysis with four Magnoliaceae Species. Forests.

[48] Wu, Z. Y. *Chinese vegetation*. 834 (Science Press, Beijing, 1980).

[49] Prince LM, Parks CR (2001). Phylogenetic relationships of Theaceae inferred from chloroplast DNA sequence data. Am. J. Bot..

[50] Yu XQ (2017). Insights into the historical assembly of East Asian subtropical evergreen broadleaved forests revealed by the temporal history of the tea family. New Phytol..

[51] Ming, T. L. & Bartholomew, B. Theaceae in *Flora of China* Vol. 12 (eds Wu Z. Y. & Raven P. H.) 366–478 (Science Press, Beijing, 2007).

[52] Rao, M. *et al.* Environmental and evolutionary drivers of diversity patterns in the tea family (Theaceae s.s.) across China. *Ecol. Evol.* **8**, 11663–11676 (2018). 10.1002/ece3.4619 PMC6303774 30598765

[53] Deng LL, Fan GS (1999). A new species of genus *Gordonia*. J. Trop. & Subtrop. Bot..

[54] Gitzendanner MA, Soltis PS, Yi TS, Li DZ, Soltis DE (2018). Plastome phylogenetics: 30 years of inferences into plant evolution. Advan. Botan. Res..

[55] Doyle JJ, Doyle JL (1987). A rapid DNA isolation procedure for small quantities of fresh leaf tissue. Phytochem. Bull..

[56] Patel RK, Jain M (2012). NGS QC Toolkit: a toolkit for quality control of next generation sequencing data. PLoS ONE.

[57] Jin JJ (2020). GetOrganelle: a fast and versatile toolkit for accurate de novo assembly of organelle genomes. Genome Biol..

[58] Greiner S, Lehwark P, Bock R (2019). OrganellarGenomeDRAW (OGDRAW) version 1.3.1: expanded toolkit for the graphical visualization of organellar genomes. Nucl. Acid. Res..

[59] Kurtz SA (2001). REPuter:the manifold applications of repeat analysis on a genomic scale. Nucl. Acid. Res..

[60] Beier S, Thiel T, Munch T, Scholz U, Mascher M (2017). MISA-web: a web server for microsatellite prediction. Bioinformatics.

[61] Frazer KA, Pachter L, Poliakov A, Rubin EM, Dubchak I (2004). VISTA: computational tools for comparative genomics. Nucl. Acid. Res..

[62] Brudno M (2003). Glocal alignment: finding rearrangements during alignment. Bioinformatics.

[63] Amiryousefi A, Hyvonen J, Poczai P (2018). IRscope: an online program to visualize the junction sites of chloroplast genomes. Bioinformatics.

[64] Darling AC, Mau B, Blattner FR, Perna NT (2004). Mauve: multiple alignment of conserved genomic sequence with rearrangements. Genome Res..

[65] Katoh K, Standley DM (2013). MAFFT multiple sequence alignment software version 7: improvements in performance and usability. Mol. Biol. Evol..

[66] Rozas J (2017). DnaSP 6: DNA sequence polymorphism analysis of large data sets. Mol. Biol. Evol..

[67] Wang, D., Zhang, Y., Zhang, Z., Zhu, J. & Yu, J. KaKs_Calculator 2.0: a toolkit incorporating gamma-series methods and sliding window strategies. *Genomics, Proteomics & Bioinformatics* **8**, 77–80 (2010). 10.1016/S1672-0229(10)60008-3 PMC5054116 20451164

[68] Tzeng YH, Pan R, Li WH (2004). Comparison of three methods for estimating rates of synonymous and nonsynonymous nucleotide substitutions. Mol. Biol. Evol..

[69] Glez-Pena D, Gomez-Blanco D, Reboiro-Jato M, Fdez-Riverola F, Posada D (2010). ALTER: program-oriented conversion of DNA and protein alignments. Nucl. Acid. Res..

[70] Deng W, Wang Y, Liu Z, Cheng H, Xue Y (2014). HemI: a toolkit for illustrating heatmaps. PLoS ONE.

[71] Wright F (1990). The 'effective number of codons' used in a gene. Gene.

[72] Sharp PM, Li WH (1986). Codon usage in regulatory genes in Escherichia coli does not reflect selection for 'rare' codons. Nucl. Acid. Res..

[73] Katoh K, Rozewicki J, Yamada KD (2019). MAFFT online service: multiple sequence alignment, interactive sequence choice and visualization. Brief Bioinform..

[74] Talavera G, Castresana J (2007). Improvement of phylogenies after removing divergent and ambiguously aligned blocks from protein sequence alignments. Syst. Biol..

[75] Zhang D (2020). PhyloSuite: An integrated and scalable desktop platform for streamlined molecular sequence data management and evolutionary phylogenetics studies. Mol. Ecol. Resour..

[76] Xia X (2017). DAMBE6: new tools for microbial genomics, phylogenetics, and molecular evolution. J. Hered..

[77] Keane TM, Creevey CJ, Pentony MM, Naughton TJ, McLnerney JO (2006). Assessment of methods for amino acid matrix selection and their use on empirical data shows that ad hoc assumptions for choice of matrix are not justified. BMC Evol. Biol..

[78] Stamatakis A (2014). RAxML version 8: a tool for phylogenetic analysis and post-analysis of large phylogenies. Bioinformatics.

[79] Miller MA, Pfeiffer W, Schwartz T (2010). Creating the CIPRES Science Gateway for inference of large phylogenetic trees *Proc*. Gatew. Comput. Environ..

