# Supplementary figures and images for: Comparative chloroplast genome and phylogenetic analyses of Chinese Polyspora
Source: Sci Rep. 2022 Sep 26;12:15984. doi: 10.1038/s41598-022-16290-4 (PMC9512918; doi:10.1038/s41598-022-16290-4)

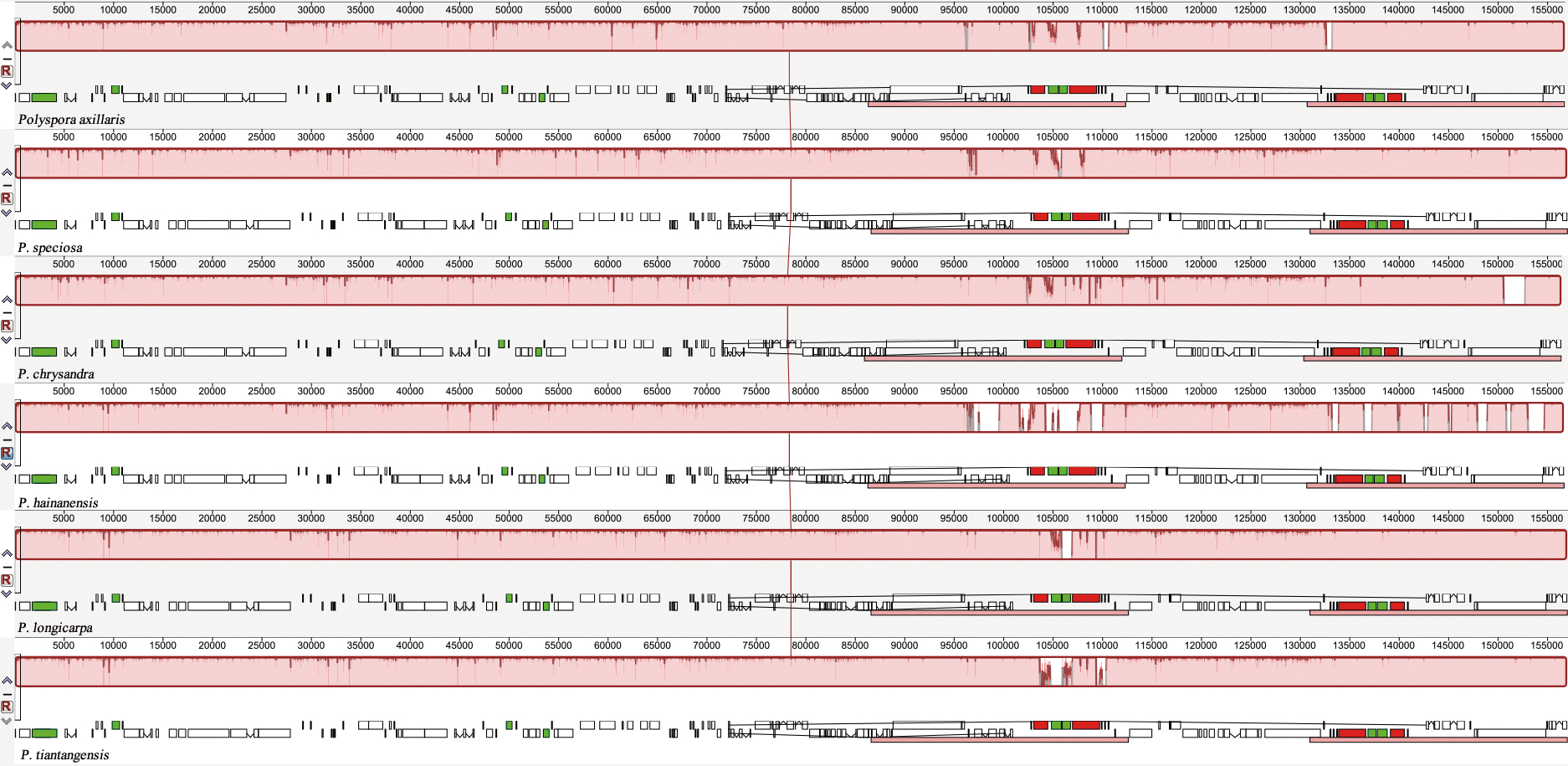

Supplement: Supplementary file 1 — Supplementary Information 1. [file 41598_2022_16290_MOESM1_ESM.tif]
