# Supplementary material for: Comparative chloroplast genome and phylogenetic analyses of Chinese Polyspora
Source: Sci Rep. 2022 Sep 26;12:15984. doi: 10.1038/s41598-022-16290-4 (PMC9512918; doi:10.1038/s41598-022-16290-4)
Supplement: Supplementary file 2 — Supplementary Information 2. [file 41598_2022_16290_MOESM2_ESM.docx]

**Supplementary figure and table titles**

Figure S1: Synteny analyses of chloroplast genomes in Chinese *Polyspora*.

Table S1: List of chloroplast genomes used for phylogenetic analysis.

Table S2: Summary of complete chloroplast genomes for Chinese *Polyspora*.

Table S3: Number of long repeats in chloroplast genomes of genus *Polyspora* from China.

Table S4: The statistic of repeat types among Chinese *Polyspora*.

Table S5: The comparison of SSRs among Chinese *Polyspora* species.

Table S6: The statistic of SSRs among Chinese *Polyspora*.

Table S7: The statistic of genetic Ka/Ks among Chinese *Polyspora*.

Table S8: The statistic of genetic Ka/Ks among *Polyspora chrysandra* and other Chinese *Polyspora*.

Table S9: Codon usage for Chinese *Polyspora* chloroplast genome.
